# TP53 mutations in triple-negative breast cancer cells confer sensitivity to ASCT2 inhibition via arginine uptake

**DOI:** 10.1038/s41419-026-08814-x

**Published:** 2026-05-21

**Authors:** Xiaodan Lyu, Yuancheng Wei, Ziyi Chen, Qianlin Zou, Jia Wang, Yi Han, Chenxi Xu, Haolin Hu, Zizhang Zhou, Shengtao Yuan, Mei Yang, Li Sun

**Affiliations:** 1 New Drug Screening and Pharmacodynamics Evaluation Center, National Key Laboratory for Multi-Target Natural Drugs, China Pharmaceutical University, Nanjing, China; 2 Laney Graduate School, Emory University, Atlanta, GA USA; 3 General Surgery, Zhongda Hospital, School of Medicine, Southeast University, Nanjing, China; 4 State Key Laboratory of Crop Biology, College of Life Sciences, Shandong Agricultural University, Tai’an, China

**Keywords:** Breast cancer, Cancer metabolism

## Abstract

Targeting dysregulated glutamine metabolism via ASCT2 inhibition has therapeutic potential in cancer, but its clinical translation is hindered by tumor metabolic heterogeneity and the lack of predictive biomarkers. This study aims to define genetic determinants of ASCT2 inhibitor sensitivity and uncover compensatory resistance mechanisms to enable precision therapeutic strategies. We systematically evaluated ASCT2 inhibitor responses across molecular subtypes of breast cancer via in vitro and in vivo models. Mechanistic studies integrated transcriptomics, metabolomics, and functional validation of candidate pathways. Using genetic and pharmacological tools, including TP53 isogenic lines and ASCT2 inhibitors, we mapped the metabolic compensation networks. This approach revealed that TP53-mutant triple-negative carcinomas were more sensitive to ASCT2 monotherapy than any other subtype. In TP53 wild-type tumors, ASCT2 inhibition triggered SLC7A3-mediated arginine uptake, destabilizing the CASTOR1-GATOR2 complex to sustain mTORC1-driven proliferation. Cotargeting ASCT2 and SLC7A3 overcame resistance in TP53 wild-type models, inducing metabolic collapse and tumor reduction. This work establishes the TP53 mutational status as a potential predictive biomarker for ASCT2 inhibitor responsiveness and defines the SLC7A3-arginine-mTORC1 axis as a targetable compensatory pathway. Therefore, we propose a genotype-guided therapeutic strategy, recommending ASCT2 monotherapy for TP53-mutant tumors and combined ASCT2 and SLC7A3 inhibition for TP53 wild-type cancers. These findings advance precision in targeting glutamine metabolism while providing a blueprint to counter adaptive resistance through rational drug combinations.

## Introduction

Glutamine metabolism has emerged as a critical regulator of oncogenesis, serving dual roles in bioenergetic provision and signaling pathway modulation [1, 2]. As the dominant glutamine transporter, ASCT2 (SLC1A5) facilitates the cellular uptake of this multifunctional metabolite, sustaining tumor proliferation through mTORC1 activation and redox homeostasis maintenance [3–5]. Because of this crucial role, ASCT2 is a compelling therapeutic target. Inhibitors such as V-9302 and C118P have shown preclinical efficacy in multiple cancers [6, 7]. However, early-phase clinical trials reveal limited efficacy. This low response rate highlights an urgent need to identify which patients are most likely to benefit [8].

The high heterogeneity of breast cancer is driven by diverse genetic alterations [9–11]. Molecular classification differences and genetic mutations constitute significant factors in determining breast cancer heterogeneity [12–14]. Notably, triple-negative breast cancer is distinct from other subtypes in terms of glutamine dependency [15], a metabolic vulnerability potentially linked to its characteristic TP53 mutations [16]. This subtype-specific metabolic reprogramming creates therapeutic opportunities but simultaneously poses challenges. Under stress conditions, wild-type TP53 plays a crucial regulatory role in tumor metabolism and intracellular amino acid pool homeostasis, manifested primarily through enhanced glycolytic activity or the activation of alternative pathways to maintain amino acid metabolic balance [17–20].

A key mechanism of resistance may involve compensatory uptake of arginine or aspartate via alternative transporters, yet this adaptive response is not well understood in clinical contexts [21, 22]. While TP53 mutations may initially be associated with glutamine addiction in BLBC, emerging evidence reveals a paradoxical metabolic resilience mechanism: CASTOR1-mediated mTORC1 hyperactivation persists in tumors [23–25]. This metabolic plasticity indicates that focusing only on TP53 status is insufficient. Therefore, a critical unmet need is to define the precise compensatory pathways that arise upon ASCT2 inhibition in different genetic contexts and to develop strategies, such as real-time metabolic monitoring and rational drug combinations, to overcome this adaptive resistance [26–29]. The expanding field of tumor metabolic reprogramming raises central questions about the barriers to effective therapy and the design of improved treatment strategies.

Collectively, cancer metabolic plasticity is particularly prominent in breast cancer, where intertumoral heterogeneity driven by TP53 status and intrinsic subtypes creates divergent metabolic dependencies [30–33]. Current precision medicine paradigms lack validated biomarkers to predict which patients derive durable benefit from glutamine metabolism-targeting agents, whereas combinatorial strategies to counteract metabolic heterogeneity remain underexplored.

Therefore, we aimed to identify TP53-mutant triple-negative breast cancer subtypes that are sensitive to ASCT2 inhibitors to identify possible indicators to guide clinical treatment. Our findings not only provide a framework for individualized precision medicine involving ASCT2 inhibitors but also reveal targetable vulnerabilities arising from therapeutic-induced metabolic stress.

## Materials and methods

### Chemicals and reagents

C118P was supplied by Sanhome Pharmaceutical Co., Ltd. (Nanjing, China). Taxol was purchased from the Yangtze River Pharmaceutical Group (Taizhou, China).

### Cells and cell culture

Human breast cancer cells (MDA-MB-231, HCC1187, MDA-MB-468, HCC1395, BT-549, and MDA-MB-436 cells) and MCF10A cells were obtained from the Shanghai Institute of Life Science at the Chinese Academy of Sciences (Shanghai, China) and the authorized distributor of the American Type Culture Collection (ATCC, Beijing, China). The authenticity of all the cell lines was confirmed through short tandem repeat (STR) analysis. The cells were grown in either DF12 medium or DMEM (supplied by Gibco, Grand Island, USA) supplemented with 10% fetal bovine serum (FBS; provided by PAN Biotech, Germany). The cells were cultured in a humidified environment in a BB15 incubator (Thermo, Germany) with 5% CO_2_ at 37 °C.

### Cell viability assay

The effects of C118P and V9302 on breast cancer cells (MDA-MB-231, HCC1187, MDA-MB-468, HCC1395, BT-549, and MDA-MB-436 cells) and MCF10A cells were determined via the MTT assay. Cell suspensions were prepared, and 2000 cells of each type were seeded into a 96-well plate. Following 24 h of incubation, the cells were treated with C118P for an additional 72 h. Afterward, 20 μL of an MTT solution (0.5 mg/mL) was added, and the cells were incubated for another 4 h, after which the medium was replaced with 150 μL of dimethyl sulfoxide (DMSO) to dissolve the formazan precipitates. The absorbance at 570 nm was measured via a universal microplate reader (Infinite M100, Tecan, Germany), from which the inhibition rates were computed via the following formula: inhibition rate (%) = (1-absorbance of the treated group/absorbance of the control group) × 100.

### Colony formation assay

The effects of treatment with C118P or V9302 on cell proliferation were assessed via colony formation assays. A total of 2000 cells were distributed in a 6-well plate for 24 h of incubation. The cells were subsequently treated with C118P or V9302 for 14 days, stabilized with a 4% formaldehyde solution, and dyed with a 0.5% solution of crystal violet. Finally, the colonies were quantified at the macroscopic level.

### Western blotting

Western blotting was performed following the methodology outlined in a prior study [34]. The following antibodies were procured from Cell Signaling Technology (MA, USA): anti-mTOR (cat# 2983), anti-p-mTOR (cat# 5536), anti-4EBP1 (cat# 9452), anti-p-4EBP1 (cat# 2855), anti-p53 (cat# 9282), and anti-p-p53 (cat# 9284). Additionally, anti-ASCT2 (cat# ab237704) and anti-mios (cat# ab202274) antibodies were purchased from Abcam (MA, USA), the anti-β-actin (cat# AC004) antibody was purchased from ABclonal Technology (Wuhan, China), and the anti-SLC7A3 (cat# NBP1-74144) was purchased from Novus (Centennial, CO, USA). Anti-rabbit HRP-conjugated IgG (cat# 7074, RRID: AB_2099233), anti-mouse HRP-conjugated IgG (cat# 7076, RRID: AB_330924) (Cell Signaling Technology) and HRP-Goat Anti-Rabbit IgG Conformation Specific Recombinant Secondary Antibody (cat# RGAR301, Proteintech, Wuhan, China) were used as secondary antibodies, and enhanced chemiluminescence reagent (Millipore) was used for detection after exposure to a Gel Doc 2000 image analyzer (Bio-Rad, CA, USA).

### Glutamine and arginine uptake assay

Following 48 h of pretreatment with C118P at a concentration of 0.05 μM, the cells (at a density of 1 × 10^5^ cells/well) were exposed to either [^3^H]-L-glutamine (400 nM, sourced from PerkinElmer) or [^3^H]-arginine in MEM (Life) for 15 min at 37 °C in the presence or absence of an inhibitor. The cells were subsequently transferred to a 96-well plate (PerkinElmer), and radioactivity was measured with a liquid scintillation counter (PerkinElmer).

### Lentivirus transfection and overexpression studies

The sequences of the small interfering RNAs (siRNAs) against human SLC1A5 and a negative control were purchased from GenePharma: siSLC1A5#1: sense 5’-GCCUUGGCAAGUACAUUCUTT-3’; siSLC1A5#2: sense 5’-GUCGACCAUAUCUCCUUGATT-3’; siSLC7A3#1: sense 5’-CUCCCACUAAUGAGCAUCUTT-3’; siSLC7A3#2: sense 5’-CCCUGAUGAUGCCUUACUATT-3’; siSLC7A3#3: sense 5’-CCUCAGGUAUCAACCUGAUTT-3’; siSLC7A3#4: sense 5’-GUCAGUUCCAUUGCUUUCUTT-3’; and negative control: sense 5’-UUCUUCCGAACGUGUCACGUTT-3’.

### Targeted metabolomics analysis

Metabolomic analysis of cells from various experimental cohorts was carried out via analytical liquid chromatography‒mass spectrometry (LC‒MS). The experimental process included biological sample collection, instrumental detection, and data analysis. Statistically significant metabolites were selected based on a univariate *p*-value < 0.05, a Variable Importance in Projection (VIP) score > 1 from the OPLS-DA model, and a fold change ≥ 1.5.

### Transcriptome analysis

RNA was collected from the cells and analyzed via library construction, Illumina HiSeq sequencing, and quantitative gene expression analysis via Cufflinks-2.2.1b. Clustering and functional enrichment were performed on the basis of the results of the differential expression analysis. Significantly different genes were identified using a threshold of an FDR below 0.05 and an absolute fold change ≥1.5.

### Chromatin immunoprecipitation (ChIP)

The binding sequence of the TP53 protein to the amino acid transporter solute-like carrier family 7, member 3 (SLC7A3) was predicted via the JASPAR database (http://jaspar.genereg.net/). After ASCT2 inhibition, ChIP experiments were performed to explore the different mechanisms by which TP53^WT^ and TP53^MT^ bind to the SLC7A3 promoter region. The ChIP experiments consisted of crosslinking, chromatin fragmentation, immunoprecipitation, DNA recovery and purification analysis, and agarose gel electrophoresis detection.

### CRISPR/Cas9

The triple-negative breast cancer cell lines MDA-MB-231 (TP53^R280K^) and HCC1395 (TP53^R175H^) were inoculated in 6-well plates for incubation overnight. The cells were incubated for an additional 48 h after transfection with the sgRNA/donor plasmid at the corresponding site. The medium was changed, puromycin (1 μg/mL) was added, and the cells were cultured for an additional 24–72 h. TP53^WT^ and TP53-null cells were obtained via monoclonal cell selection and gene identification.

### Nude mouse xenograft study

Female BALB/c athymic nude mice, aged 5–6 weeks and weighing between 18 and 22 grams, were procured from the Model Animal Research Center at Nanjing University. A total of 2 × 10^6^ MDA-MB-231 cells, each with various TP53 mutations, were injected into the subcutaneous tissue of the axilla. The tumors were allowed to grow until their volume reached between 300 and 500 mm^3^, at which point they were excised and sectioned into small pieces. The tumor tissue fragments were then subcutaneously transplanted into the nude mice. Animals were randomly assigned to either the treatment or control group (*n* = 6 per group) using a computer-generated random number sequence. No blinding was performed in this study. Mice received the following treatments: C118P (50 mg/kg) via tail vein injection, V-9302 (37.5 mg/kg) via intraperitoneal injection, paclitaxel (TAX, 10 mg/kg) via tail vein injection, or an equal volume of normal saline (control). The mice were euthanized 21 days after administration, and the tumor tissues were resected and assessed. The tumor volume (TV) was calculated via the following formula: TV (mm^3^) = A/2×B^2^, where A represents the longest diameter of the tumor, and B represents the shortest diameter. The relative tumor volume (RTV) was calculated with the following formula: RTV = V_t_/V_0_, where V_t_ represents the TV on day t, and V_0_ represents the TV on day 0. The Animal Care and Control Committee of China Pharmaceutical University guided the animal care and surgical procedures.

### Organoid culture and viability assay

Patient-derived organoid (PDO) models were generated from selected breast cancer tissues. The processing step involved isolating single cells or small clusters, followed by 3D culture in Matrigel to support organoid formation and growth. To assess the dynamic response of PDOs to treatment, PDOs were cultured in 48-well plates. Following treatment, bright-field images were captured at 24, 48, 72, and 96-h intervals to document morphological changes. Cell viability was quantified using the CellTiter-Glo® 3D Cell Viability Assay (Promega). The resulting luminescence, proportional to the ATP content and thus the number of viable cells, was recorded using a microplate reader. Dose-response curves were generated by plotting luminescence signal against drug concentration.

### Statistical analyses

The data were analyzed with GraphPad Prism software (GraphPad Software Inc., La Jolla, CA). Data are presented as mean ± SD from at least three independent experiments. Data with a single independent variable across multiple levels were analyzed by one-way analysis of variance. For data involving two independent variables, two-way analysis of variance was employed. All analyses of variance were followed by appropriate post-hoc tests to correct for multiple comparisons, with Tukey’s test or Šídák’s test used for all pairwise comparisons or Dunnett’s test for comparisons against a single control group. *P* < 0.05 was considered statistically significant (*P < 0.05, **P < 0.01, ***P < 0.001); ns indicates no significant difference.

### Ethics approval and consent to participate

All animal experimental procedures were conducted in accordance with the relevant guidelines and were approved by China Pharmaceutical University with Institutional Review Board (IRB) number 2022-10-023. This study involving human patient-derived organoids was approved by the Independent Ethics Committee (IEC) for Clinical Research of Zhongda Hospital, Affiliated to Southeast University (No.: 2023ZDSYLL056-P01). The tissues were procured through surgical resection, with comprehensive consent from each patient. All procedures were conducted in accordance with the ethical principles of the Declaration of Helsinki.

## Results

### TP53 mutations sensitize triple-negative breast cancer cells to ASCT2 inhibition

**Fig. 1:**
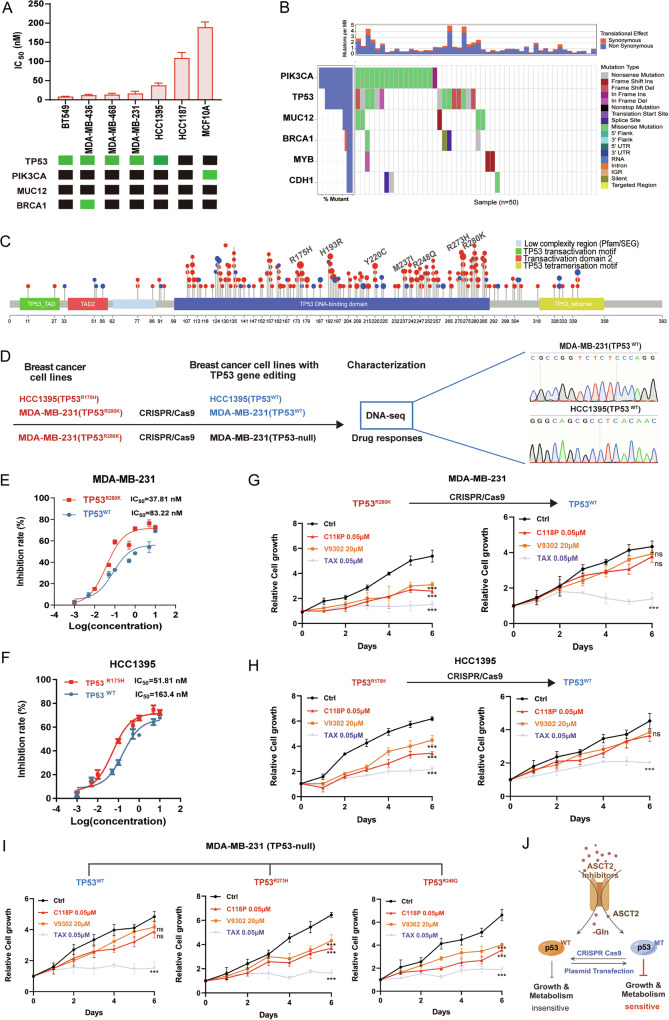
Differential sensitivity of TP53^WT^ and TP53^MT^ breast cancer cells to ASCT2 inhibitors. **A** Cell viability after C118P treatment was analyzed via MTT assays. **B** Well-known mutated oncogenes in breast cancer. **C** Lollipop plot displaying the mutation distribution and protein domains of the TP53 gene in breast cancer cells; the recurrent hotspots are labeled. **D** MDA-MB-231 and HCC1395 cells were edited via CRISPR/Cas9, and TP53 status was confirmed via Sanger sequencing. **E**, **F** The IC_50_ values of MDA-MB-231 and HCC1395 cells were determined via MTT assays. *n* = 3. **G**, **H** Breast cancer cell growth after treatment with the ASCT2 inhibitors C118P (0.05 μM) and V9302 (20 μM). *n* = 3. **I** TP53-null triple-negative breast cancer cells were transfected with TP53-mutant (R273H and R248Q) or TP53-wild-type plasmids. The growth of TP53^WT^ and TP53^MT^ MDA-MB-231 cells was detected after treatment with the ASCT2 inhibitors C118P and V9302. *n* = 3. **J** Schematic diagram illustrating our experimental strategy for establishing cellular models with distinct TP53 mutation statuses through CRISPR/Cas9-mediated gene editing and plasmid transfection approaches. By systematically comparing the sensitivity of these genetically modified cells to ASCT2 inhibitors, we demonstrate that compared with their TP53 wild-type counterparts, TP53-mutant or TP53-null cells exhibit significantly enhanced drug responsiveness. The error bars represent the means ± SD of at least three independent experiments; statistical significance was determined by one-way ANOVA with Dunnett’s multiple comparisons test. *P < 0.05; **P < 0.01; ***P < 0.001 vs. Ctrl.

Glutamine addiction varies widely among breast cancer subtypes, yet reliable predictive biomarkers for glutamine-targeted therapies remain underexplored. ASCT2 (SLC1A5), a key glutamine transporter, mediates intracellular glutamine accumulation, and its inhibition results in substantial glutamine depletion. To investigate the therapeutic potential of targeting ASCT2, we systematically evaluated the responses of a panel of breast cancer cell lines to ASCT2 inhibitors (C118P and V9302). Consistent with the heterogeneous glutamine dependence observed in triple-negative breast cancer, we observed a different pattern of responses. A subset of triple-negative cell lines was highly sensitive to ASCT2 inhibition (IC_50_ < 50 nM), whereas others maintained proliferation via glutamine-independent mechanisms (Figs. 1A and S1A–D).

To explore potential determinants of this differential sensitivity, we integrated drug response data with genomic alterations. Mutation profiling revealed that TP53 was among the frequently altered genes in our panel (Fig. 1B). Correlation analyses demonstrated that intrinsic sensitivity to ASCT2 inhibitors was not associated with ASCT2 or TP53 protein expression levels (Fig. S1E), suggesting that TP53 mutational status, rather than protein abundance, is a more relevant predictor of response.

Analysis of the TCGA database revealed R175H, R273H, R248Q, and R280K as the most frequent TP53 mutation sites in breast cancer (Fig. 1C). To investigate the functional impact of TP53 loss or mutation, we generated TP53-mutant (targeting R175H and R280K) and TP53-null breast cancer cell lines via the CRISPR/Cas9 system (Fig. 1D). Notably, TP53 mutation (R175H, R280K) significantly reduced the IC_50_ of C118P compared with that of TP53^WT^ cells (Fig. 1E, F), suggesting that TP53 loss or mutation enhances sensitivity to C118P. Furthermore, the growth curves revealed that C118P and V9302 inhibition decreased in TP53^WT^ cells, and the same results were obtained from the colony formation assays (Figs. 1G, H and S2A, B). To further validate this finding, we transfected TP53-null breast cancer cells with exogenous plasmids expressing TP53^R273H^, TP53^R248Q^, or TP53^WT^. In the same genetic background, TP53-mutant and TP53-null cells were more sensitive to C118P inhibition than TP53^WT^ cells were (S2C, D). Compared with those in TP53-mutant cells, growth curve assays consistently revealed reduced inhibitory effects of C118P and V9302 on TP53^WT^ cells (Fig. 1I). To validate the functional role of mutant p53 in conferring sensitivity, we reintroduced a TP53 ^R273H^ expression plasmid into TP53 wild-type MDA-MB-231 cells. Conversely, re-expression of mutant p53 significantly increased cellular sensitivity to both C118P and V-9302 treatment, thereby successfully rescuing the drug-sensitive phenotype (Fig. S2E, F).

The combined data indicate that compared with TP53^WT^ cells, TP53-mutant (R280K, R175H, R273H, and R248Q) and TP53-null triple-negative breast cancer cells exhibit increased sensitivity to ASCT2 inhibitors (Fig. 1J).

### TP53 mutation status determines therapeutic vulnerability to ASCT2 inhibition in triple-negative breast cancer in vivo

**Fig. 2:**
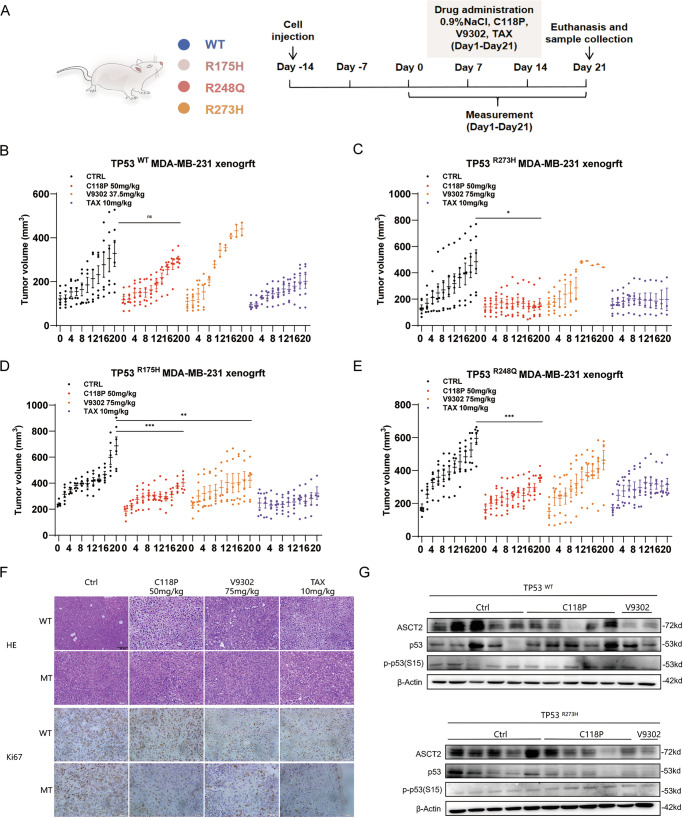
Effect of mutations in TP53 on sensitivity to ASCT2 inhibitors in vivo. **A** Schematic diagram of the animal experiment: Xenograft models were established using cells with TP53^WT^ or TP53^MT^, followed by group allocation, administration of ASCT2 inhibitors, and sample collection for analysis. **B**–**E** Growth of TP53^WT^ and TP53^MT^ MDA-MB-231 xenograft tumors after treatment with ASCT2 inhibitors. *n* = 6. **F** Histopathological and proliferative analysis. Representative H&E staining images show altered tissue morphology in response to inhibitor treatment. Immunohistochemical staining for Ki67 demonstrates treatment effects on tumor cell proliferation (scale bars: 50 µm). **G** Molecular validation of treatment efficacy. ASCT2 and p53 expression were detected via Western blotting. The error bars represent the means ± SD; statistical significance was determined by one-way ANOVA with Dunnett’s multiple comparisons test. *P < 0.05; **P < 0.01; ***P < 0.001.

Previous studies have shown that solid tumors exhibit regional nutrient heterogeneity, with glutamine depletion in the hypoxic core compared with that in the well-perfused periphery [35]. Since glutamine deficiency is proposed to restrict tumor growth, we hypothesized that inhibiting ASCT2 would suppress tumor progression by exacerbating intracellular glutamine deprivation. To test this hypothesis, we established xenograft models by injecting MDA-MB-231 triple-negative breast cancer cells harboring TP53 mutations (R273H, R175H, R248Q) or wild-type TP53 into nude mice (Fig. 2A). Compared with vehicle-treated control tumors, TP53-mutant xenografts significantly impaired tumor growth, whereas TP53 wild-type tumors showed no significant response (Figs. 2B–E and S3A–C), recapitulating the effects observed in TP53^R280K^ models [6]. Immunohistochemistry analysis further revealed reduced Ki67 expression in ASCT2-inhibited TP53^MT^ tumors (Figs. 2F and S3D), indicating suppressed proliferation.

Mechanistically, ASCT2 inhibition upregulated p-p53 in TP53^WT^ tumors (Figs. 2G and S3E). These findings suggest that TP53 is required for the cellular response to glutamine deprivation, potentially through compensatory pathways. Together, these data demonstrate that TP53 mutations dictate the sensitivity of triple-negative breast cancer to ASCT2 inhibitors, highlighting a genotype-dependent vulnerability in glutamine metabolism.

### TP53 mutation status dictates metabolic adaptation to ASCT2 inhibition

**Fig. 3:**
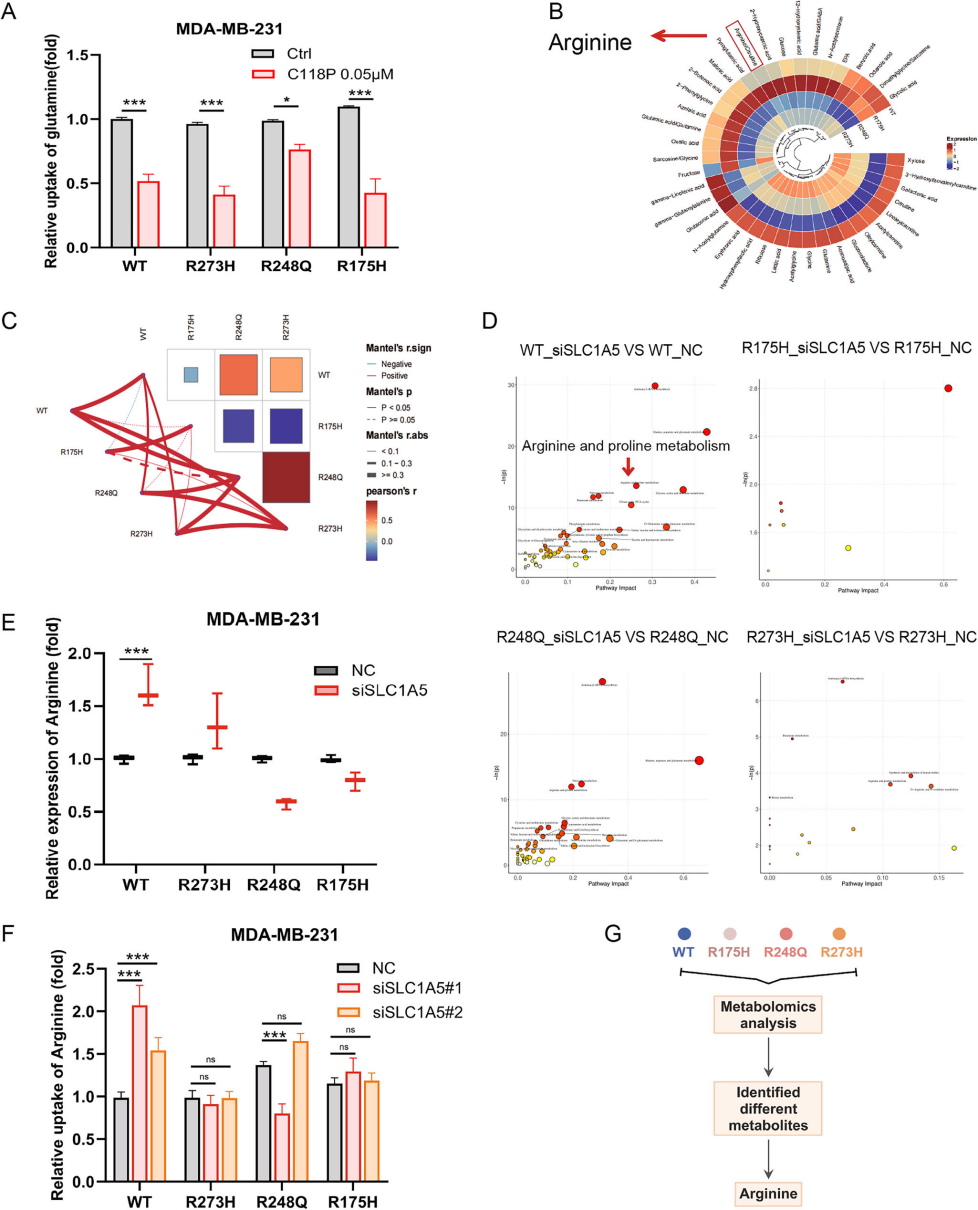
Effects of TP53 mutations on intracellular amino acid homeostasis in triple-negative breast cancer cells. **A** Glutamine uptake was quantified after 48 h of treatment with C118P. **B** Fold changes in the metabolite concentrations in siSLC1A5-knockdown cells relative to those in negative control cells. **C** Correlation analysis of amino acid levels between the TP53 mutant and wild-type groups. **D** Significantly altered metabolic pathways identified by OPLS-DA modeling of differentially abundant metabolites. **E** Intracellular arginine levels after ASCT2 inhibition. **F** Arginine uptake rates were measured via isotope tracing following 48 h of siSLC1A5 treatment. **G** Schematic workflow integrating metabolomics-based metabolite screening and validation of arginine as a key regulator. The error bars represent the means ± SD; statistical significance was determined by two-way ANOVA with Dunnett’s multiple comparisons test. *P < 0.05; **P < 0.01; ***P < 0.001.

To investigate how TP53 mutations modulate metabolic responses to ASCT2 inhibition, we first examined whether the mutations alter ASCT2-mediated glutamine uptake. We observed that baseline glutamine uptake did not differ between TP53^WT^ cells and TP53^MT^ cells (R273H, R175H, R248Q) in the absence of treatment, indicating that the TP53 status does not interfere with ASCT2 transporter activity. C118P treatment robustly suppressed glutamine uptake in all tested cell lines, including both TP53^WT^ and TP53^MT^ (R273H, R175H, R248Q) variants (Fig. 3A). Next, we performed metabolomic profiling to map the global metabolic changes induced by ASCT2 knockdown. Comparative analysis revealed distinct metabolic signatures between TP53^WT^ and TP53^MT^ cells, with 40 metabolites showing the most significant alterations (Figs. 3B and S4A–D). Notably, hierarchical clustering demonstrated partial overlap between the TP53^WT^ and TP53^R273H^/TP53^R248Q^ clusters (Fig. 3C), suggesting residual metabolic similarity in this specific mutant. Our pathway analysis revealed that arginine/proline metabolism was the most significantly enriched in TP53^WT^ cells. The upregulation of this pathway indicates a key compensatory adaptation, which establishes a logical metabolic foundation for the observed resistance (Fig. 3D and S3E, F). To validate this finding, we quantified arginine levels across genotypes and observed TP53^WT^-specific accumulation of intracellular arginine upon ASCT2 knockdown (Fig. 3E). Mechanistically, isotope tracing experiments revealed that ASCT2 inhibition significantly enhanced arginine uptake in TP53^WT^ cells (Fig. 3F). Collectively, by analyzing metabolomic alterations in wild-type versus mutant cells, we identified differentially abundant metabolites and further established arginine as the pivotal metabolic determinant through targeted validation (Fig. 3G).

### TP53 activation induced by ASCT2 inhibition upregulates SLC7A3

**Fig. 4:**
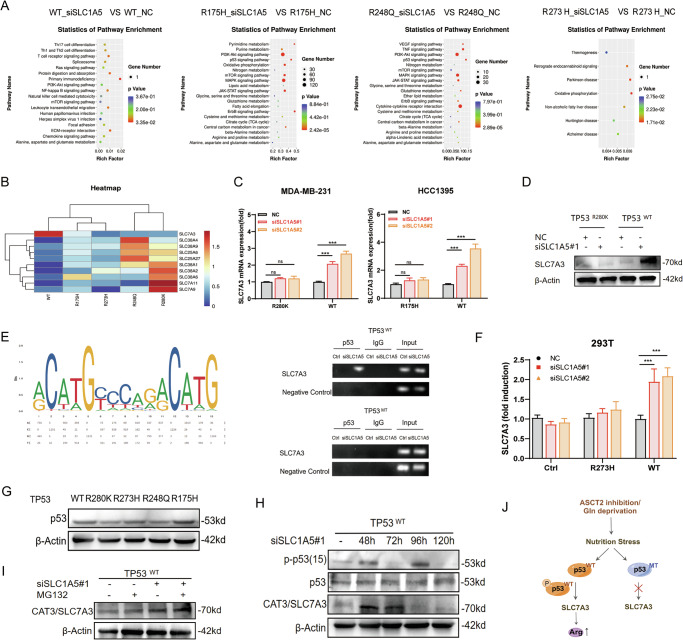
TP53 drives SLC7A3 expression to metabolically compensate for ASCT2 inhibition in triple-negative breast cancer cells. **A** Pathway enrichment analysis (GeneAnalytics) of differentially expressed genes identified by RNA sequencing. **B** qPCR validation of transcriptome-derived candidate genes in TP53 wild-type (WT) versus mutant (MT) cells. **C**, **D** SLC7A3 mRNA and protein levels were quantified by qRT‒PCR and Western blotting in TP53^WT^ and TP53^MT^ cells. **E** Computational prediction of TP53 binding motifs on the SLC7A3 promoter, including the sequence logo, position frequency matrix. ChIP assays confirming TP53 occupancy of the SLC7A3 promoter in ASCT2-inhibited TP53^WT^ cells (with an anti-p53 antibody and IgG as a control). **F** Luciferase reporter assays comparing SLC7A3 promoter activity in TP53^WT^ and TP53^MT^ cells. **G** TP53 protein expression in the TP53^WT^ and TP53^MT^ MDA-MB-231 cell lines. **H** Western blot analysis of TP53 and SLC7A3 in TP53^WT^ cells after siSLC1A5-mediated ASCT2 knockdown. **I** SLC7A3 protein levels in TP53^WT^ cells treated with siSLC1A5 or the proteasome inhibitor MG132 (10 µM, 6 h). **J** Schematic model illustrating TP53-mediated transcriptional upregulation of SLC7A3 upon ASCT2 inhibition, which enhances arginine uptake to compensate for glutamine deprivation. The error bars represent the means ± SD; statistical significance was determined by two-way ANOVA with Dunnett’s multiple comparisons test. *P < 0.05; **P < 0.01; ***P < 0.001.

To further elucidate the metabolic response of TP53 to ASCT2 inhibition, using knockdown of ASCT2 in TP53^WT^ and TP53^MT^ cells, we performed transcriptomic profiling to map the consequent changes in gene expression. Genes whose expression was upregulated in TP53^WT^ cells but whose expression was unchanged or decreased in TP53^MT^ cells upon ASCT2 inhibition were identified (Fig. S5A). Pathway enrichment analysis highlighted their associations with arginine metabolism and mTOR signaling (Fig. 4A). Interestingly, we found that SLC7A3, a plasma membrane transporter for cationic amino acids, was upregulated upon ASCT2 inhibition in TP53^WT^ cells (Figs. 4B and S5B–E). Given the TP53^WT^-specific induction of SLC7A3 under glutamine deprivation, we hypothesized that SLC7A3 might be a direct transcriptional target of TP53. To test this hypothesis, we first compared SLC7A3 expression in TP53^WT^ and TP53^MT^ cells. SLC7A3 upregulation was more pronounced in the WT group after ASCT2 knockdown (Fig. 4C, D). Consistent with these findings, ChIP assays using an anti-p53 antibody revealed enrichment of TP53 binding to the SLC7A3 promoter region in ASCT2-inhibited TP53^WT^ cells (Fig. 4E). Luciferase reporter assays further demonstrated that TP53 activation enhances SLC7A3 promoter activity (Fig. 4F).

Previous studies linked glutamine deprivation to TP53 activation [36]. While TP53 missense mutations did not alter total TP53 protein levels, ASCT2 inhibition triggered TP53 phosphorylation in TP53^WT^ cells, indicating functional activation (Fig. 4G–I). Collectively, these data establish that TP53 activation under ASCT2 inhibition transcriptionally upregulates SLC7A3, thereby increasing arginine uptake to compensate for glutamine deficiency (Fig. 4J).

### Cotargeting ASCT2 and SLC7A3 synergistically inhibits tumor growth in TP53^WT^ triple-negative breast cancer

**Fig. 5:**
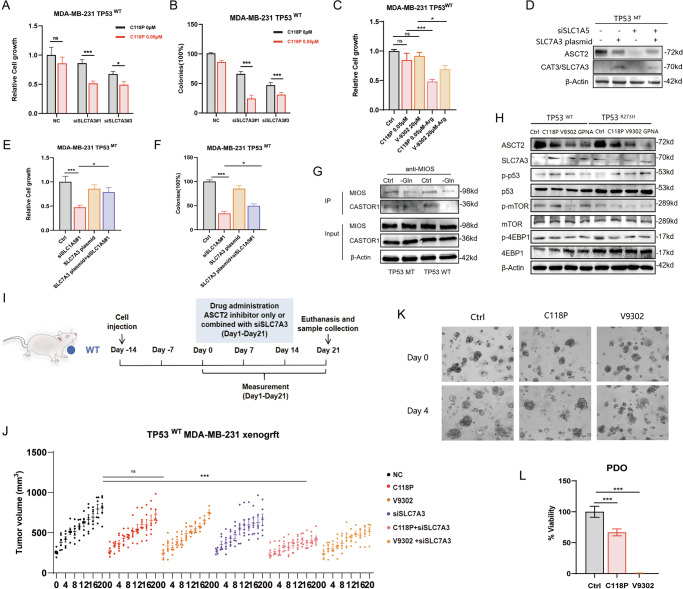
TP53 affects cell sensitivity to ASCT2 inhibitors via SLC7A3. **A**, **B** Cell viability was assessed after treatment with the ASCT2 inhibitor C118P or siSLC7A3 in TP53^WT^ MDA-MB-231 cells. **C** Cell viability was assessed after ASCT2 inhibitor (C118P) or arginine deprivation treatment in TP53^WT^ MDA-MB-231 cells. **D** Western blot analysis was performed to verify the transfection efficiency of SLC7A3 overexpression in TP53^MT^ MDA-MB-231 cells. **E**, **F** Cell viability was assessed after TP53^MT^ MDA-MB-231 cells were treated with siSLC1A5 or SLC7A3 plasmid. **G** The binding of MIOS to CASTOR1 was detected via co-IP. **H** Protein expression of ASCT2, p53, p-p53, CAT3/SLC7A3, mTOR, p-mTOR, 4EBP1, p-4EBP1, and β-actin in TP53^WT^ and TP53^MT^ breast cancer cells after treatment with ASCT2 inhibitors. **I** The animal experiment process is shown in the figure. **J** Growth of TP53^WT^ MDA-MB-231 xenograft tumors after treatment with ASCT2 inhibitors or siSLC7A3. **K** Representative images showing organoid morphological changes before and after ASCT2 inhibition. Scale bars, 50 μm. **L** Organoid viability in response to ASCT2 inhibition as measured by ATP assay. The error bars represent the means ± SD of at least three independent experiments; statistical significance was determined by one-way ANOVA or two-way ANOVA with Dunnett’s test or Tukey’s test or Šídák’s test. *P < 0.05; **P < 0.01; ***P < 0.001.

C118P inhibited TP53^WT^ cell growth, and SLC7A3 knockdown increased the sensitivity of these cells to C118P (Figs. 5A, B and S6A–D). These same results were obtained with TP53^WT^ cells after arginine deprivation (Figs. 5C and S6E). Overexpression of SLC7A3 in TP53^MT^ cells and simultaneous knockdown of SLC1A5 significantly inhibited cell growth (Figs. 5D–F and S6F, G). Additionally, in TP53^WT^ cells, ASCT2 deletion inhibited the binding of CASTOR1 to GATOR2 (Fig. 5G). Next, we studied the signaling activity of mTORC1. As expected, downstream mTORC1 signaling was activated in TP53^WT^ cells but not in TP53^MT^ cells (Figs. 5H and S6H). Similarly, SLC7A3 knockdown enhanced the efficacy of the ASCT2 inhibitors C118P and V9302 in the TP53^WT^ in vivo model (Figs. 5I, J and S6I–K).

To validate our findings in a clinically relevant model, we tested ASCT2 inhibitors in PDO from triple-negative breast cancer. Consistent with prior models, drug response was stratified by TP53 status: ASCT2 inhibition significantly suppressed proliferation specifically in PDOs harboring TP53 mutations (Figs. 5K, L and S6L). This concordant response across cell lines, xenografts, and now patient-derived organoids strongly implicates TP53 mutation as a key determinant of sensitivity, providing a multi-layered preclinical rationale for its clinical investigation.

**Fig. 6:**
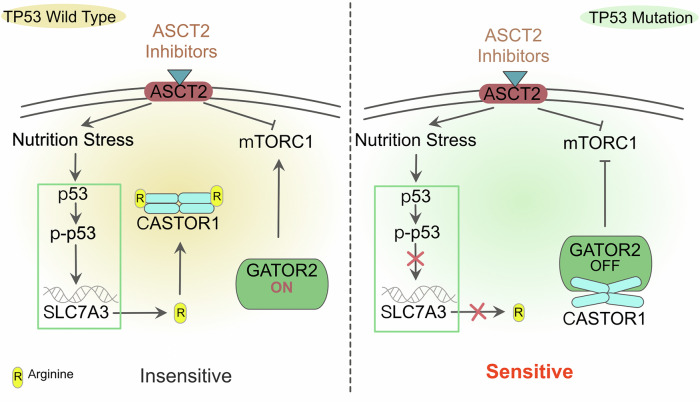
Schematic model of TP53-dependent metabolic heterogeneity in response to asct2 inhibition in triple-negative breast cancer. In TP53-wild-type tumors (left panel), ASCT2 inhibition induces glutamine deprivation, activating TP53 to transcriptionally upregulate the arginine transporter SLC7A3. Increased arginine influx disrupts CASTOR1-GATOR2 interactions, resulting in mTORC1 activation. Conversely, in TP53-mutant tumors (right panel), loss of functional TP53 abrogates SLC7A3 induction, leading to arginine scarcity. This stabilizes the CASTOR1-GATOR2 complex, maintaining mTORC1 suppression.

This study addresses the clinical challenge of metabolic heterogeneity hindering ASCT2 inhibitor efficacy by identifying TP53-mutant triple-negative breast cancer as a responsive subtype. The mechanism involves SLC7A3-mediated arginine uptake triggered by ASCT2 inhibition. In TP53-wild-type tumors, this disrupts the CASTOR1-GATOR2 complex to activate a compensatory mTORC1 pathway, whereas this axis is non-functional in TP53-mutant counterparts. Consequently, TP53-mutant tumors exhibit heightened vulnerability to ASCT2 monotherapy, whereas TP53-wild-type cancers require combinatorial SLC7A3 blockade. These findings establish a genotype-guided therapeutic paradigm for targeting glutamine addiction while elucidating metabolic compensation mechanisms that inform rational therapeutic combinations (Fig. 6).

## Discussion

The development of targeted therapies against metabolic vulnerabilities in cancer remains limited by tumor heterogeneity and microenvironmental interactions. Our study reveals a genotype-defined metabolic dependency in triple-negative breast cancer, demonstrating that the TP53 mutational status dictates therapeutic responses to ASCT2 inhibition through an SLC7A3‒mTORC1 regulatory axis.

Our data position TP53 as the key regulator of the metabolic compensation that occurs when ASCT2 is blocked. In TP53 wild-type tumors, ASCT2 inhibition triggers TP53-dependent transcriptional upregulation of SLC7A3. This compensatory response elevates intracellular arginine levels, which destabilizes the CASTOR1-GATOR2 complex to activate mTORC1 as an adaptive survival mechanism (Fig. 6). In contrast, TP53-mutant tumors cannot mount this SLC7A3 response. Thus, TP53 status helps explain the variable clinical responses to ASCT2 inhibitors and emerges as a candidate predictive biomarker.

The contrast in therapeutic response highlights the need for genotype-guided approaches. Whereas TP53^MT^ tumors show marked sensitivity to ASCT2 monotherapy, TP53^WT^ tumors resist treatment via SLC7A3-mediated arginine utilization. Cotargeting ASCT2 and SLC7A3 in TP53^WT^ models overcomes this resistance through dual blockade of glutamine and arginine metabolism, which disrupts mTORC1 signaling. This strategy is consistent with emerging concepts of synthetic metabolic lethality [37–40], exemplified in KRAS-mutant cancers where NOP56 and mTOR co-inhibition bypasses adaptive rewiring [41]. Similarly, the GLUT1/ALDOB/G6PD axis has emerged as a target in chemotherapy-resistant pancreatic cancer [42]. Our work extends this paradigm to TP53-altered cancers, proposing combination regimens tailored to the genetic context.

The interplay between genetic drivers and metabolic plasticity is illustrated by the dual role of TP53 in stress adaptation. Unlike HER2-positive breast cancers, which upregulate glycolysis to evade targeted therapies [43], TP53^WT^ tumors appear to exploit SLC7A3 to sustain mTORC1 activity under nutrient stress, a mechanism analogous to BRAF-mutant melanomas that leverage oxidative phosphorylation during MAPK inhibition [44–46]. Importantly, TP53 mutations seem to disrupt this adaptive hierarchy, rendering tumors reliant on more inflexible metabolic programs. By linking CASTOR1-GATOR2 destabilization to TP53 loss, our data suggest a mechanistic basis for subtype-specific vulnerabilities. Further studies examining site-specific transcriptional controls of SLC7A3 will help clarify its precise mechanistic role in this adaptive response.

Based on our findings, we recommend including TP53 genotyping to select patients in future clinical trials of ASCT2 inhibitors. In TP53^MT^ triple-negative breast cancers, these drugs may work well as single agents. For TP53^WT^ tumors, monitoring metabolic adaptation through glutamine or arginine tracking with PET imaging could indicate the appropriate timing to introduce an SLC7A3 inhibitor. Early data also point to possible synergy between ASCT2 and mTORC1 blockers in TP53^WT^ models. Together, these insights now chart a clear path forward by validating TP53 as a predictive biomarker through retrospective biospecimen analysis and prospective clinical testing. Ultimately, success in future clinical trials will depend on our ability to balance maximal synthetic lethality in TP53^WT^ tumors against the safety of normal tissues.

We compared the responses of breast cancer subtypes to ASCT2 inhibition and found that TP53-mutant triple-negative tumors are uniquely sensitive. This work shows that differential sensitivity hinges on amino acid metabolism, specifically arginine flux. Mechanistically, we link TP53 status to SLC7A3-dependent adaptation, which suggests a practical strategy, for example, using combined ASCT2 and SLC7A3 inhibitors in TP53 wild-type tumors. Together, these findings define genotype-specific metabolic vulnerabilities and resistance mechanisms, providing a framework for more precise therapy in breast cancer.

## Conclusions

This work identifies ASCT2 inhibitors as a genotype-specific treatment for TP53-mutant triple-negative breast cancer, where they show stronger antitumor effects than in TP53-wild-type tumors. We put forward a mechanistic hypothesis involving a compensatory SLC7A3–arginine–mTORC1 axis, which could account for earlier inconsistent clinical findings and offers a molecular basis for stratifying patients and designing drug combinations. By connecting genetic changes to metabolic reliance, these insights help steer the clinical development of ASCT2-targeted therapies and move precision oncology forward for hard-to-treat breast cancer subtypes.

## Supplementary information

Supplementary materials

Figure S1

Figure S2

Figure S3

Figure S4

Figure S5

Figure S6

Supplementary table

uncropped western blots

## Data Availability

All relevant data are available within the paper and its supplementary materials. The sequencing data generated in this study have been deposited in the NCBI Sequence Read Archive under accession codes PRJNA1371347 and PRJNA1390874. The data that support the findings of this study are available from the corresponding author upon reasonable request.
