# Supplementary material for: TP53 mutations in triple-negative breast cancer cells confer sensitivity to ASCT2 inhibition via arginine uptake
Source: Cell Death Dis. 2026 May 21;17(1):640. doi: 10.1038/s41419-026-08814-x (PMC13365229; doi:10.1038/s41419-026-08814-x)
Supplement: Supplementary file 1 — Supplementary materials [file 41419_2026_8814_MOESM1_ESM.docx]

**Figure legends**

Fig. S1 Effects of an ASCT2 inhibitor on the proliferation of breast cancer cells. (A-D) Growth curves and colony formation ability of breast cancer cells treated with the ASCT2 inhibitors C118P (0.05 μM) or V9302 (20 μM). (E) Western blot analyses of the TP53 protein and ASCT2 in triple-negative breast cancer cells. The error bars represent the means ± SD of at least three independent experiments; statistical significance was determined by Student’s t-test. **P<0.01; ***P<0.001.

Fig. S2 Differences in drug sensitivity between TP53^WT^ and TP53^MT^ breast cancer cells. (A-B) Growth and colony formation ability of breast cancer cells treated with the ASCT2 inhibitors C118P (0.05 μM) and V9302 (20 μM). (C) Cells were constructed via plasmid transfection. (D) An MTT assay was performed to analyze the IC_50_ values of TP53^WT^ and TP53^MT^ in MDA-MB-231 cells. (E-F) Cell growth was detected after treatment with ASCT2 inhibitors (C118P and V9302). TP53^WT^ MDA-MB-231 cells were either transfected with TP53 ^R273H^ plasmid or served as a transfection control. The error bars represent the means ± SD of at least three independent experiments; statistical significance was determined by two-way ANOVA with Sidak's multiple comparisons test. *P<0.05; **P<0.01; ***P<0.001.

Fig. S3 In vivo response to ASCT2 inhibition is dependent on TP53 status. (A) Tumor growth of MDA-MB-231 cells in a xenograft model. (B) Kaplan‒Meier survival curve for xenograft tumor model mice. (C) Tumor weights of TP53^WT^ and TP53^MT^ MDA-MB-231 cells in a xenograft model after treatment with ASCT2 inhibitors; paclitaxel (TAX) was used as the positive control. (D) Image quantification in Figure 2F was performed. (E) The statistical data of the Western blots in Figure 2G are shown. The error bars represent the means ± SD; statistical significance was determined by one-way ANOVA or two-way ANOVA with Dunnett's multiple comparisons test. *P<0.05; **P<0.01; ***P<0.001.

Fig. S4 Metabolomic analysis of intracellular amino acid levels in TP53^WT^ versus TP53^MT^ triple-negative breast cancer cells. (A-D) Fold change in the differential expression of metabolites in the negative control group relative to the siSLC1A5 group. (E-F) Summary of changes in the arginine metabolism pathway.

Fig. S5 Transcriptomic analysis of genetic alterations. (A) Heatmap of siSLC1A5 in basal-like breast cancer cells in vitro. SPIN ordered expression matrix of genes differentially expressed between the siControl and siSLC1A5 groups. The colors indicate the relative expression after each gene was standardized. Dendrogram showing hierarchical clustering of data from the transcriptome analysis of duplicate negative control and siSLC1A5 treated samples. (B) Integrated bioinformatics analysis identifies TP53-associated target. (C) The expression of the amino acid transporter SLC in TP53^WT^ and TP53^MT^ breast cancer cells was analyzed via qRT-PCR. (D) Pathway enrichment analysis. (E) The expression of the amino acid transporter SLC7A1-3 in TP53^WT^ and TP53^MT^ breast cancer cells was analyzed via qRT-PCR. The error bars represent the means ± SD; statistical significance was determined by two-way ANOVA with Dunnett's multiple comparisons test. *P<0.05; **P<0.01; ***P<0.001.

Fig. S6 Effects of SLC7A3 in TP53^WT^ or TP53^MT^ cells on C118P drug susceptibility. (A) Western blot analysis was performed to verify the transfection efficiency of SLC7A3 knockdown in TP53^WT^ MDA-MB-231 cell lines. (B) SLC7A3 mRNA expression was knocked down in the MDA-MB-231 cell line. (C) Growth curves of TP53^WT^ MDA-MB-231 cells treated with the ASCT2 inhibitor C118P (0.05 μM) and siSLC7A3. (D) Colony formation of TP53^WT^ MDA-MB-231 cells treated with the ASCT2 inhibitor C118P (0.05 μM) and siSLC7A3. (E) Growth curves of TP53^WT^ MDA-MB-231 cells treated with the ASCT2 inhibitor C118P (0.05 μM) and deprived of arginine. (F) Growth curves of TP53^MT^ MDA-MB-231 cells treated with siSLC1A5 and SLC7A3 plasmids. (G) Colony formation of TP53^MT^ MDA-MB-231 cells treated with siSLC1A5 and SLC7A3 plasmids. (H) The statistical data of the Western blots in Figure 5H are shown. (I) Tumor growth of TP53^WT^ xenograft model. (J) Images of H&E staining and immunohistochemical staining for Ki67. Scale bars, 50 μm. (K) Image quantification of Ki67 in Figure S6J was performed. (L) Images of H&E staining in PDO were shown. Scale bars, 50 μm.

The error bars represent the means ± SD; statistical significance was determined by one-way ANOVA or two-way ANOVA with Dunnett's multiple comparisons test. *P<0.05; **P<0.01; ***P<0.001.
