# Supplementary figures and images for: TP53 mutations in triple-negative breast cancer cells confer sensitivity to ASCT2 inhibition via arginine uptake
Source: Cell Death Dis. 2026 May 21;17(1):640. doi: 10.1038/s41419-026-08814-x (PMC13365229; doi:10.1038/s41419-026-08814-x)

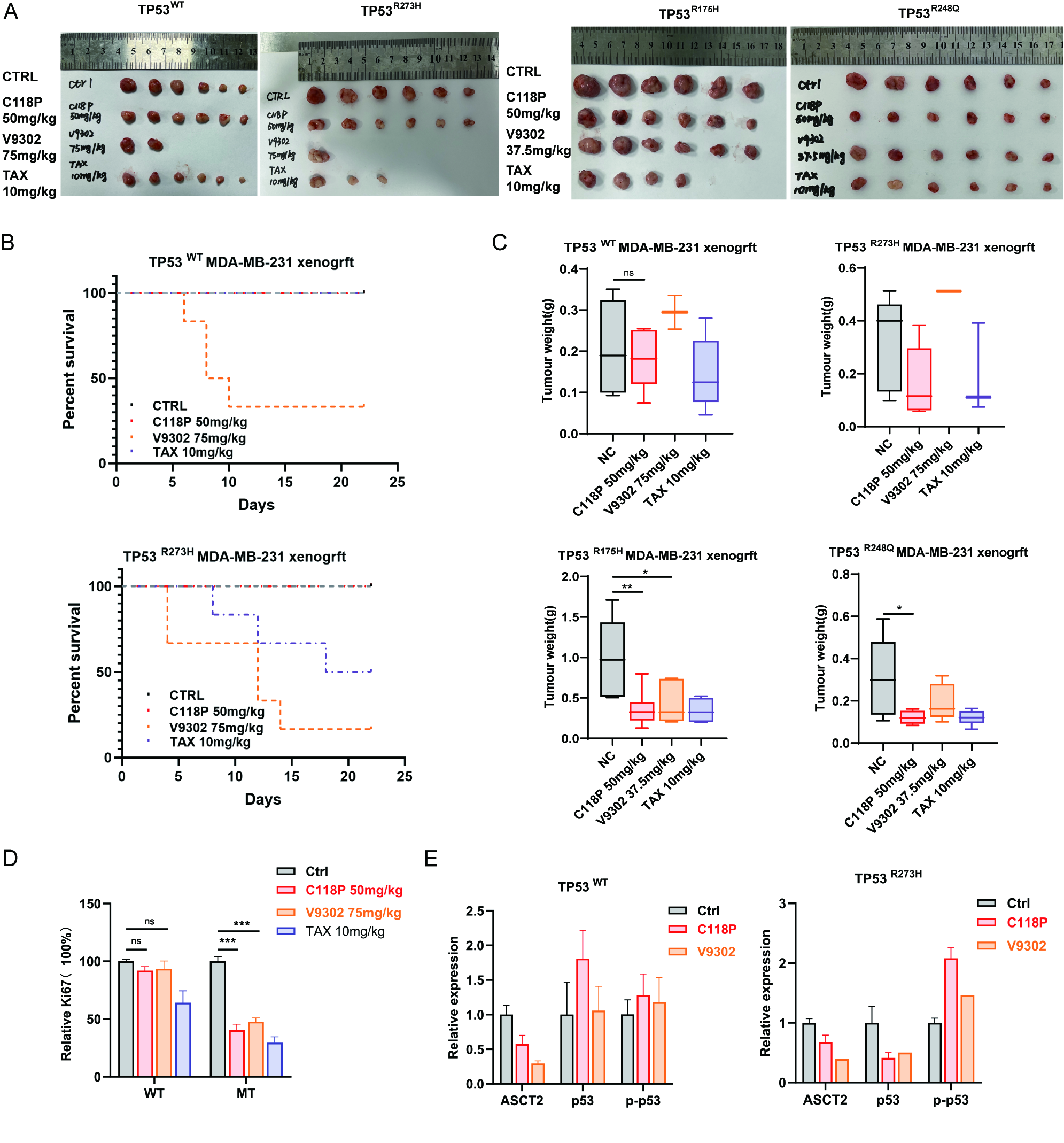

Supplement: Supplementary file 4 — Figure S3 [file 41419_2026_8814_MOESM4_ESM.tif]

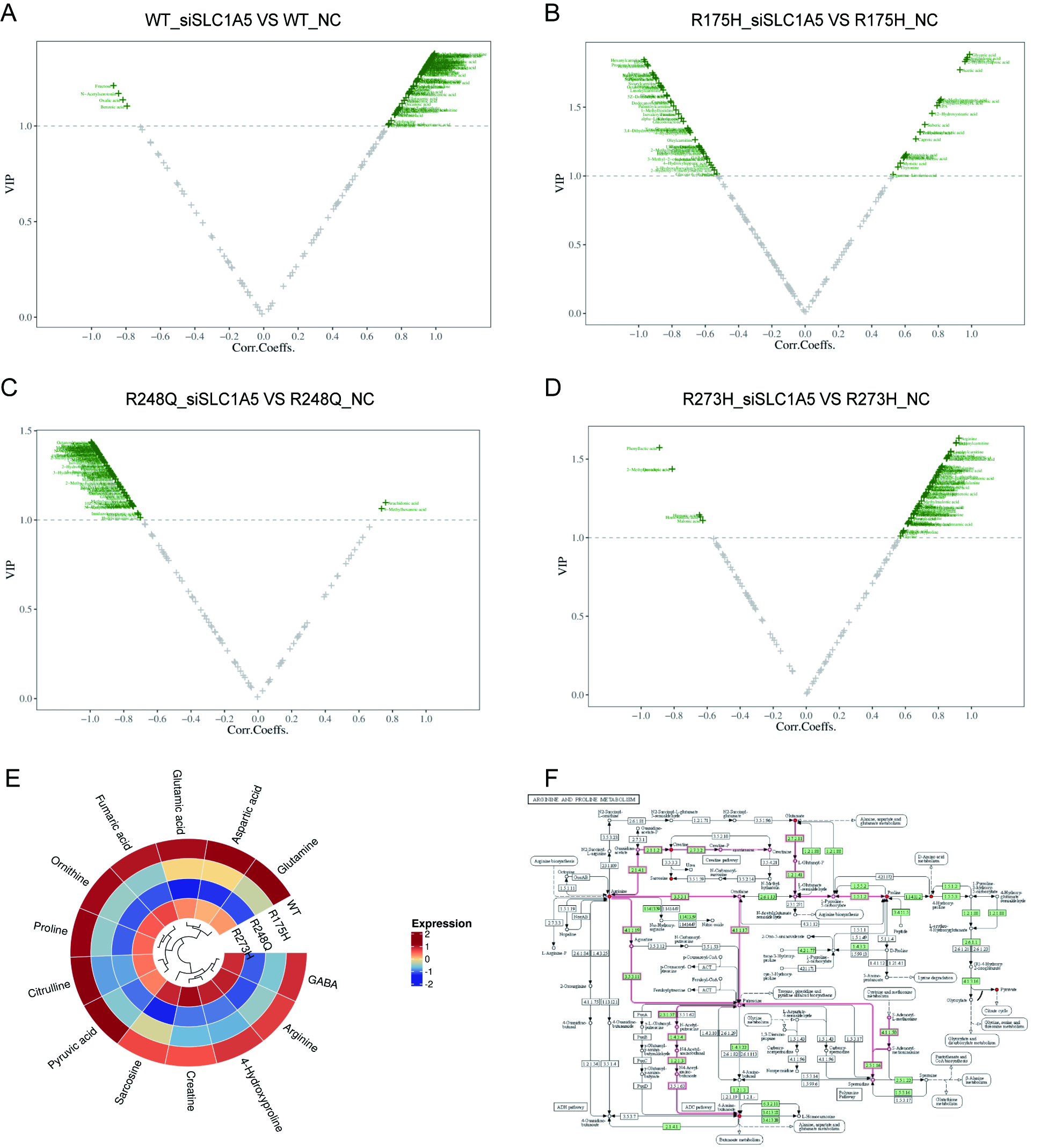

Supplement: Supplementary file 5 — Figure S4 [file 41419_2026_8814_MOESM5_ESM.tif]

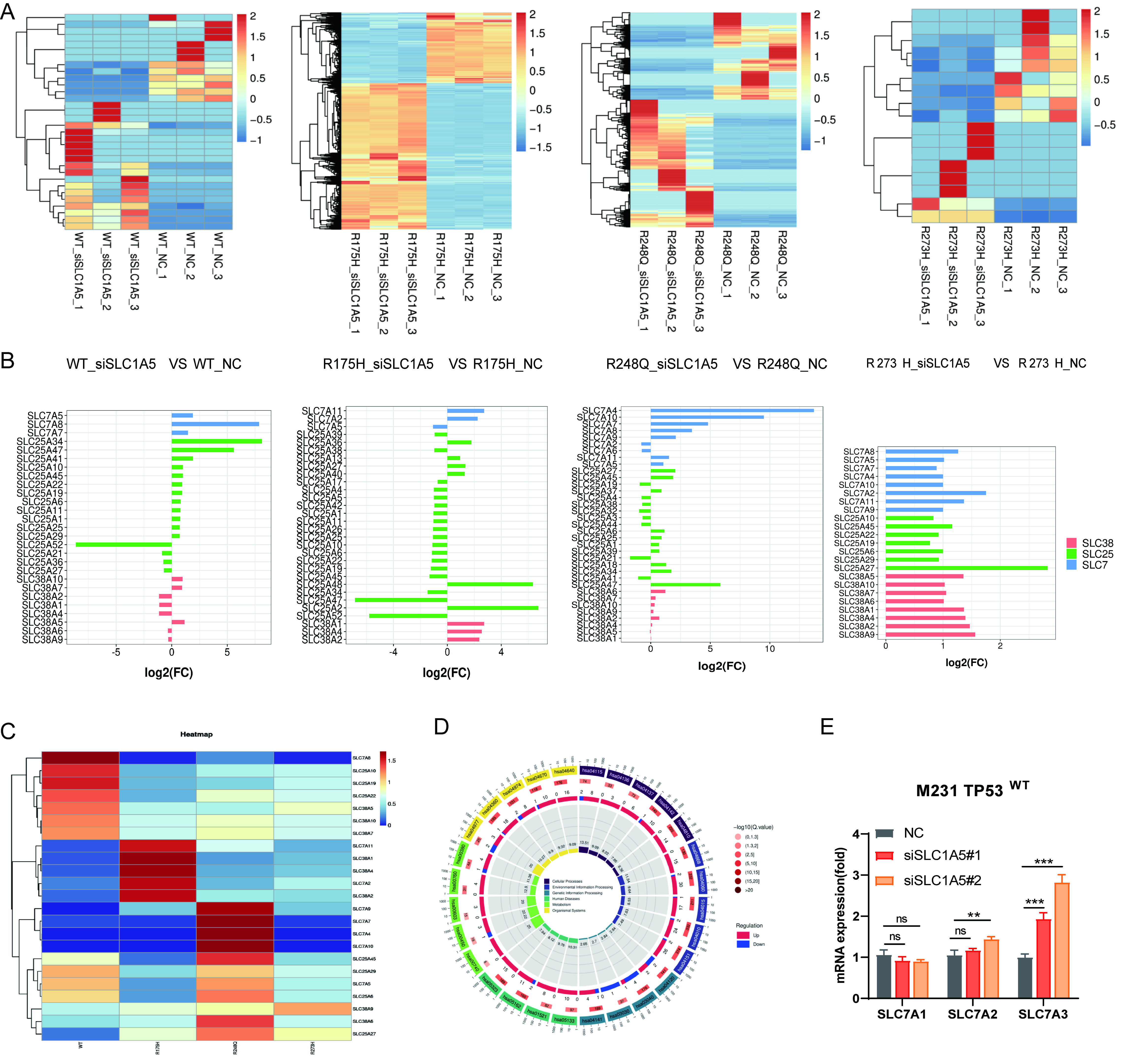

Supplement: Supplementary file 6 — Figure S5 [file 41419_2026_8814_MOESM6_ESM.tif]

Fig 2G

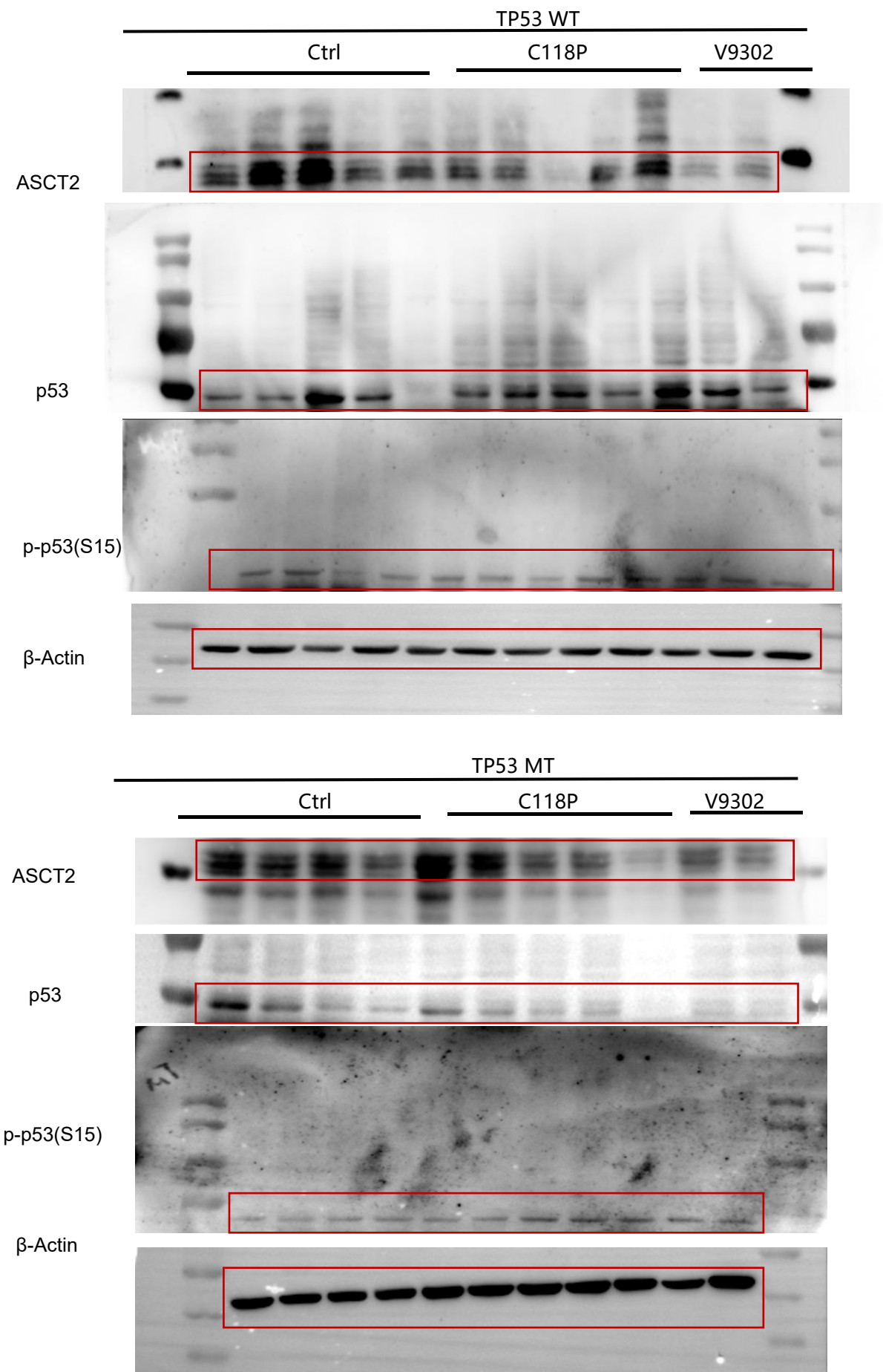

Fig 4D

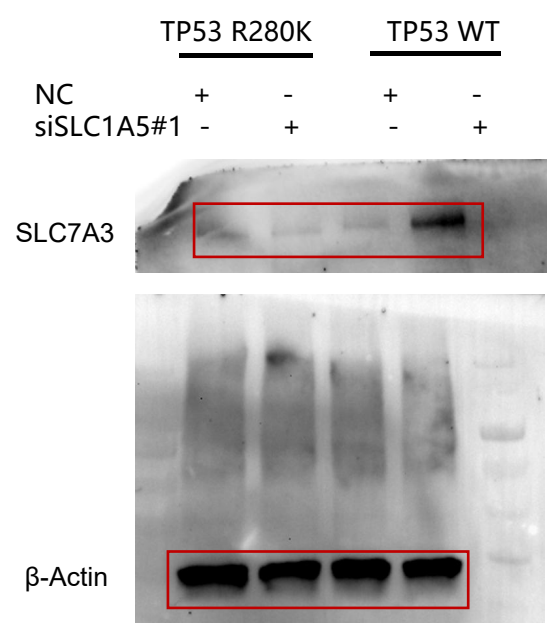

Fig 4G

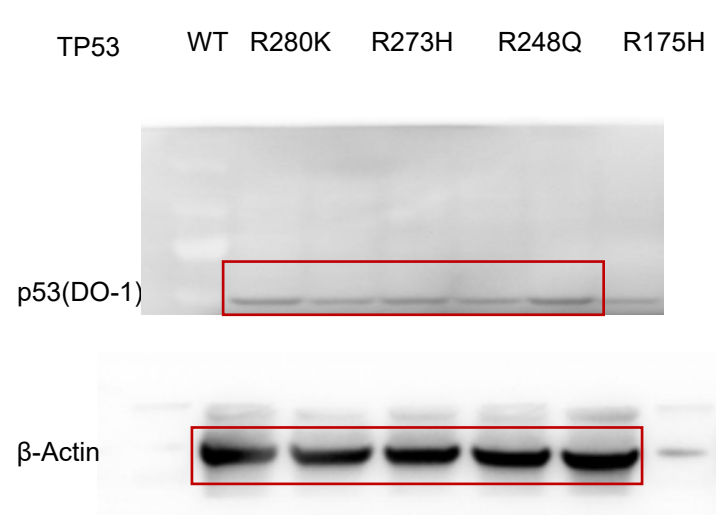

Fig 4H

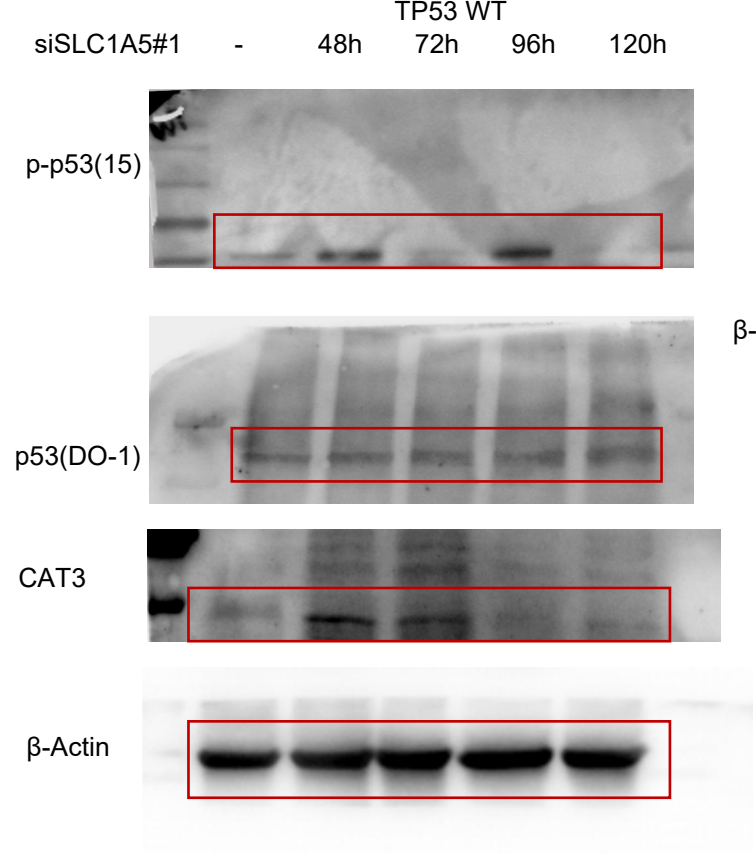

Fig 4I

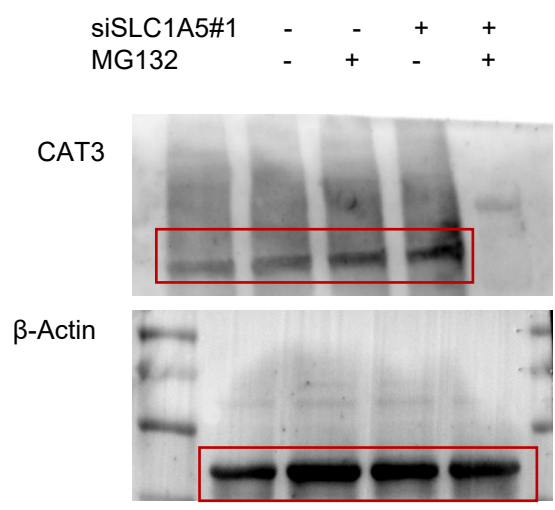

Fig 5D

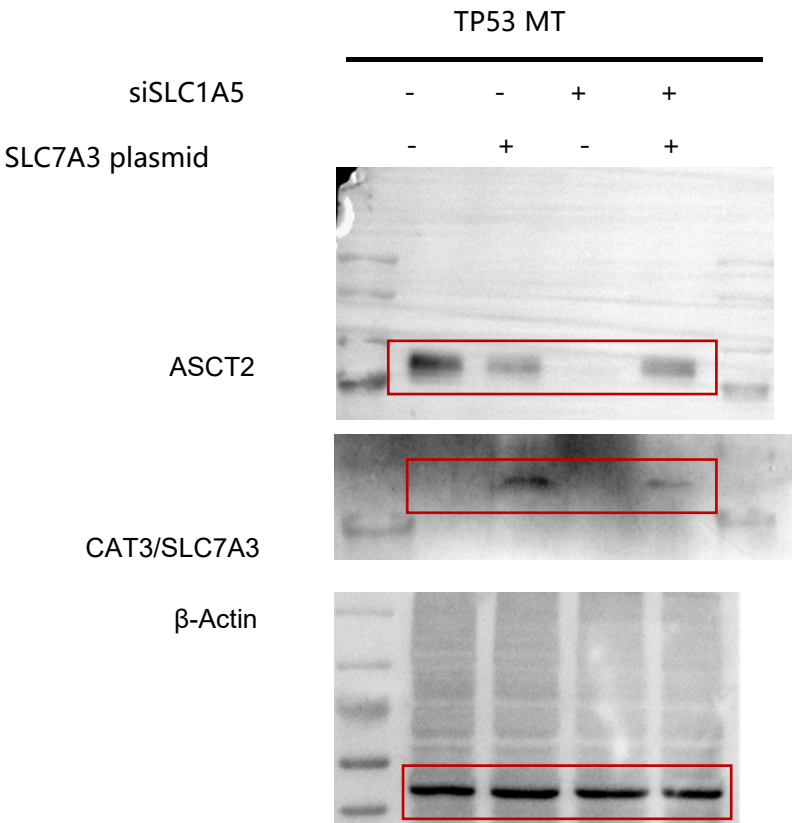

Fig 5G

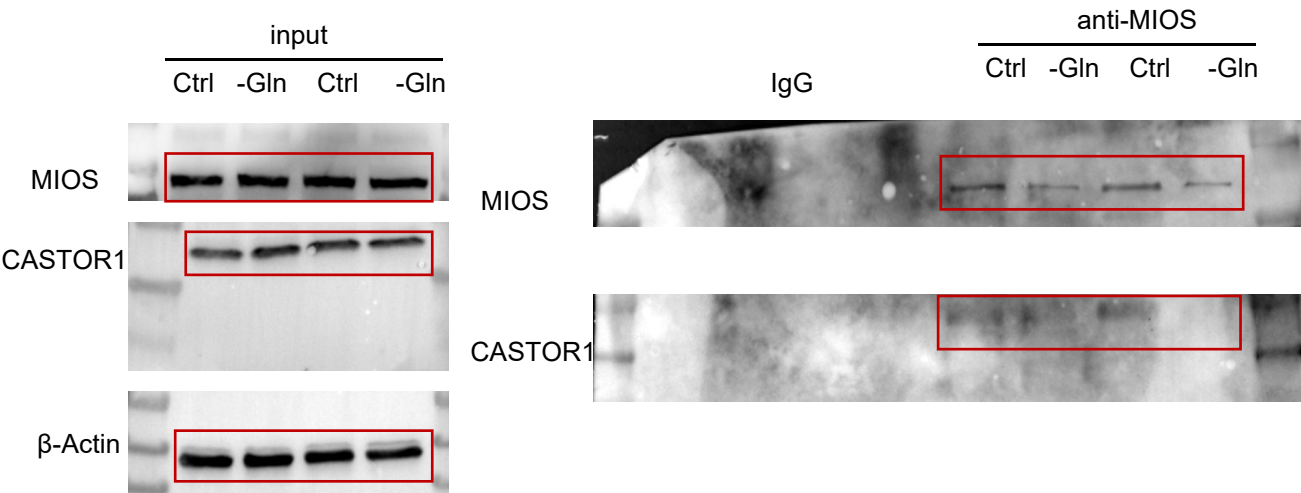

Fig 5

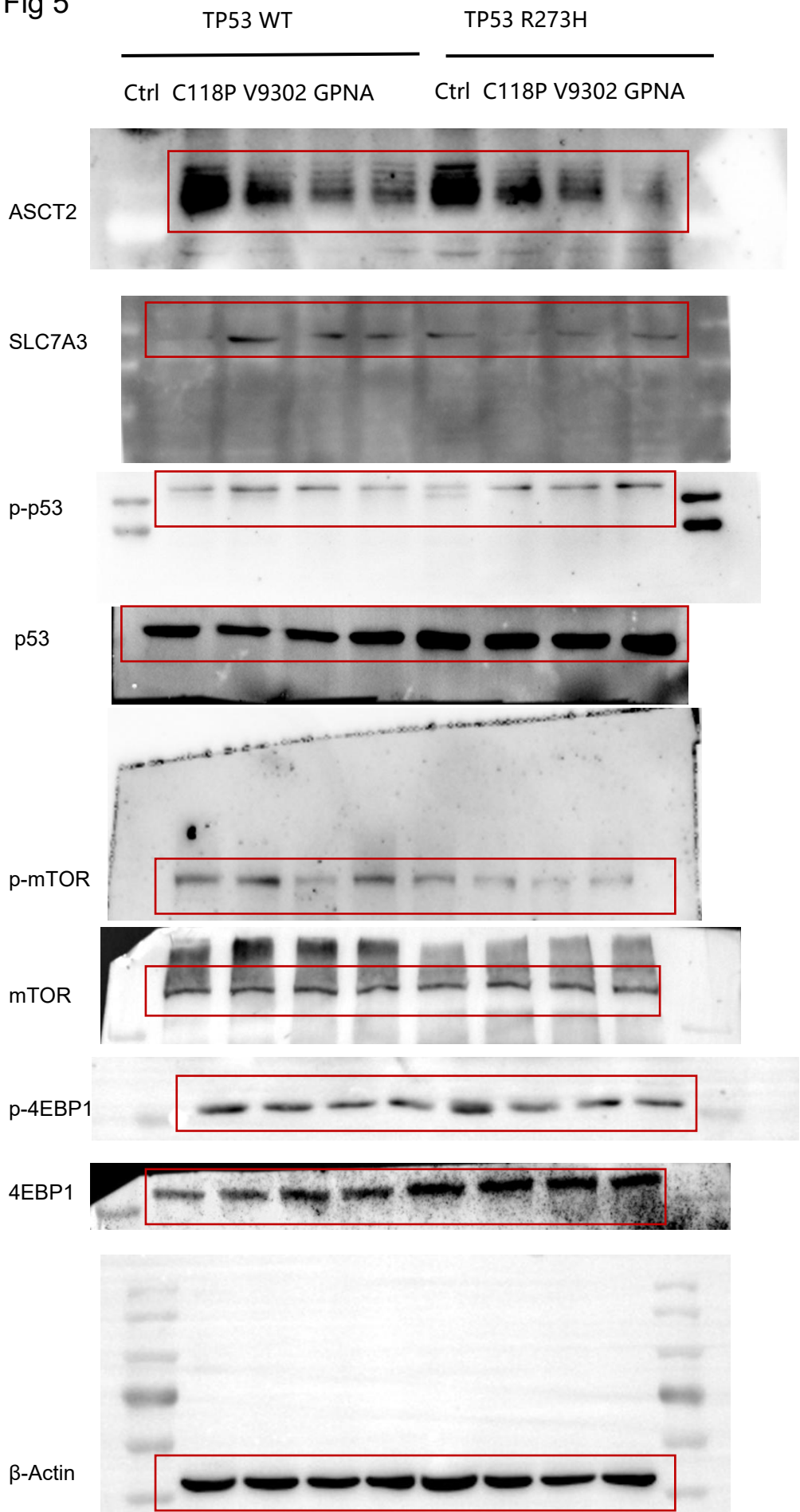

Fig S1E

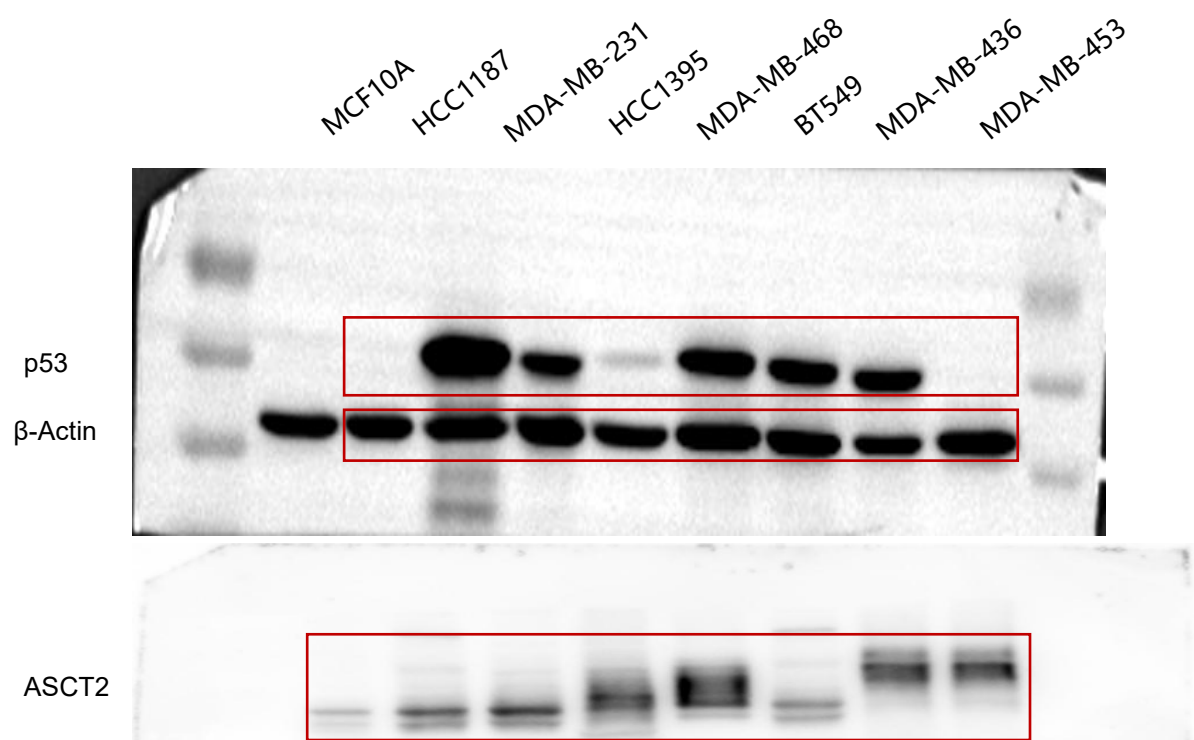

Fig S6A

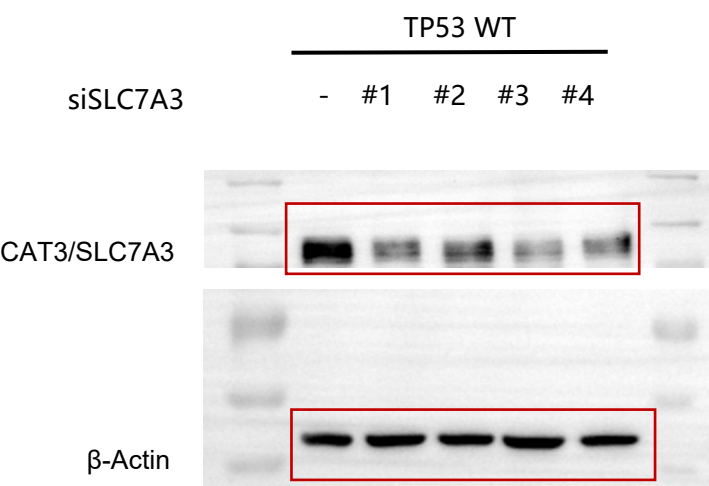

Supplement: Supplementary file 9 — uncropped western blots [file 41419_2026_8814_MOESM9_ESM.pdf]
