# Supplementary material for: TP53 mutations in triple-negative breast cancer cells confer sensitivity to ASCT2 inhibition via arginine uptake
Source: Cell Death Dis. 2026 May 21;17(1):640. doi: 10.1038/s41419-026-08814-x (PMC13365229; doi:10.1038/s41419-026-08814-x)
Supplement: Supplementary file 8 — Supplementary table [file 41419_2026_8814_MOESM8_ESM.docx]

**Supplementary Table 1:** Primer sequence for qPCR

| Gene |  | Sequence |
| --- | --- | --- |
| SLC7A11 | F | GTCTGGGTGGAACTCCTCAT |
|  | R | CTCCAGCTGACACTCATGCTA |
| GCN2 | F | AAATGCCCACCTACCTATCCA |
|  | R | CCTCCCCACAGTGTTTCTTGG |
| SLC38A9 | F | CAGTGGTCGAGTCTCCTTTTC |
|  | R | TCCCGGCACTTGGACAAATC |
| 18S | F | CAGCCACCCGAGATTGAGCA |
|  | R | TAGTAGCGACGGGCGGTGTG |
| SLC1A5 | F | TCCTCTTCACCCGCAAAAACCC |
|  | R | CCACGCCATTATTCTCCTCCAC |
| TP53 | F | CAGCACATGACGGAGGTTGT |
|  | R | TCATCCAAATACTCCACACGC |
| SLC7A3 | F | CACTCAACTCCATCCCCACT |
|  | R | AGTGGACATAGAGAGTGCCG |
| SLC7A5 | F | CCGTGAACTGCTACAGCGT |
|  | R | CTTCCCGATCTGGACGAAGC |
| SLC38A2 | F | ACCGCAGCCGTAGAAGAATG |
|  | R | GCCAGACGGACAATGAGAAGAA |
| SLC7A8 | F | AGGCTGGAACTTTCTGAATTACG |
|  | R | ACATAAGCGACATTGGCAAAGA |
| SLC7A9 | F | TGGGCACCATCATTGGCTC |
|  | R | GGCCTCCATCAGGTAGGGAT |
| SLC7A11 | F | F: GTCTGGGTGGAACTCCTCAT |
|  | R | R: CTCCAGCTGACACTCATGCTA |
| SLC7A2 | F | CCTTATGGCTTTACGGGAACG |
|  | R | CGAGGAGGTAGTACGGCATCA |
| SLC7A4 | F | CTGGCAGCCCTATGCTATGC |
|  | R | CCGATGATGTATTCGAGGAGAAC |
| SLC38A1 | F | CACCACAGGGAAGTTCGTAATC |
|  | R | CATCCACGTACCAGGCTGAAA |
| SLC38A4 | F | ATTACACCCACCGCAATCCTG |
|  | R | CGTCCGGGAGTTGAATACAAAG |
| SLC38A6 | F | GCTTTTGACAGTCCCTCTAATCC |
|  | R | TTGATGTACTGGCACCAACTAC |
| SLC38A9 | F | CAGTGGTCGAGTCTCCTTTTC |
|  | R | TCCCGGCACTTGGACAAATC |
| SLC25A10 | F | ACCTGCTCAAGGTGCATCTG |
|  | R | CAGGGAGTAGGTCATCTGTCTG |
| SLC25A45 | F | CTGAGACGCAGAGTGTACCAG |
|  | R | GCACTGTTGATGGTGACCC |
| SLC25A22 | F | GCCAGCCAAGCTCATCAATG |
|  | R | GAGGCAGTCGGACATGCTC |
| SLC25A19 | F | GTGAGCCCAAGGTCTATAATACG |
|  | R | TCTTTCCTTCGGCTGGTATGG |
| SLC25A27 | F | CCCGAGCGAGCAAATTCCTA |
|  | R | ACTACGTGTCTGTAAATGGCG |
| SLC25A6 | F | GCAACCTTGCCAACGTCATTC |
|  | R | CCGCAAAGTACCTCCAGAACT |
| MUTPRIMER1 | F | CACAAACATGCACCTCAAAGCTGTTCCGTCCC |
|  | R | TGAGGTGCATGTTTGTGCCTGTCCTGGGAGAG |
| MUTPRIMER2 | F | CGGTCTTTCCCAGGACAGGCACAAACACGCAC |
|  | R | TGTCCTGGGAGAGACCGGCGCACAGAGGAAG |
| MUTPRIMER3 | F | GCAGTGCCTCACAACCTCCGTCATGTGCTGTG |
|  | R | AGGTTGTGAGGCACTGCCCCCACCATGAGCGC |

**Supplementary Table 2:** The target sequences are shown as follows:

|  | sequences |
| --- | --- |
| sgRNA-1 | CACCGCAGTCACAGCACATGACGG |
| sgRNA-2 | AAACCCGTCATGTGCTGTGACTGC |
| sgRNA-3 | CACCTGGGAAAGACCGGCGCACAG |
| sgRNA-4 | AAACCTGTGCGCCGGTCTTTCCCA |
